# Effects of *NFKB1* and *NFKBIA* Gene Polymorphisms on Susceptibility to Environmental Factors and the Clinicopathologic Development of Oral Cancer

**DOI:** 10.1371/journal.pone.0035078

**Published:** 2012-04-11

**Authors:** Chiao-Wen Lin, Yih-Shou Hsieh, Chung-Han Hsin, Chun-Wen Su, Chien-Huang Lin, Lin-Hung Wei, Shun-Fa Yang, Ming-Hsien Chien

**Affiliations:** 1 Institute of Biochemistry and Biotechnology, Chung Shan Medical University, Taichung, Taiwan; 2 Department of Medical Research, Chung Shan Medical University Hospital, Taichung, Taiwan; 3 School of Medicine, Chung Shan Medical University, Taichung, Taiwan; 4 Department of Otolaryngology, Chung Shan Medical University Hospital, Taichung, Taiwan; 5 Institute of Medicine, Chung Shan Medical University, Taichung, Taiwan; 6 Graduate Institute of Medical Sciences, College of Medicine, Taipei Medical University, Taipei, Taiwan; 7 Department of Oncology, National Taiwan University Hospital, Taipei, Taiwan; 8 Wan Fang Hospital, Taipei Medical University, Taipei, Taiwan; 9 Graduate Institute of Clinical Medicine, College of Medicine, Taipei Medical University, Taipei, Taiwan; Ospedale Pediatrico Bambino Gesú, Italy

## Abstract

**Background:**

Oral cancer, which is the fourth most common cancer in Taiwanese men, is associated with environmental carcinogens. The possibility that genetic predisposition in nuclear factor-kappa B (NF-κB)-signaling pathways activation is linked to the development of oral squamous cell carcinoma (OSCC) requires investigation. The current study examines associations between polymorphisms within promoter regions of *NFKB1* encoding NF-κB1 and *NFKBIA* encoding IkappaBalpha (IκBα) with both the susceptibility to develop OSCC and the clinicopathological characteristics of the tumors.

**Methodology/Principal Findings:**

Genetic polymorphisms of *NFKB1* and *NFKBIA* were analyzed by a real-time polymerase chain reaction (real-time PCR) for 462 patients with oral cancer and 520 non-cancer controls. We found that *NFKB1* −94 ATGG1/ATGG2, −94 ATGG2/ATGG2, and the combination of −94 ATGG1/ATGG2 and ATGG2/ATGG2 genotypes *NFKBIA* −826 T (CT+TT) and −881 G (AG+GG) allelic carriages, were more prevalent in OSCC patients than in non-cancer participants. Moreover, we found that *NFKB1* or *NFKBIA* gene polymorphisms seem to be related to susceptibility to develop oral cancer linked to betel nut and tobacco consumption. Finally, patients with oral cancer who had at least one −519 T allele of the *NFKBIA* gene were at higher risk for developing distant metastasis (*P*<.05), compared with those patients CC homozygotes.

**Conclusions:**

Our results suggest that *NFKB1* −94 ATTG2, *NFKBIA* −826 T, and −881 G alleles are associated with oral carcinogenesis. The combination of *NFKB1* or *NFKBIA* gene polymorphisms and environmental carcinogens appears related to an increased risk of oral cancer. More importantly, the genetic polymorphism of *NFKBIA −*519 might be a predictive factor for the distal metastasis of OSCC in Taiwanese.

## Introduction

Oral squamous cell carcinoma (OSCC), a common malignant cancer of the head and neck region, is the fourth most common cancer among men and the sixth leading cause of cancer deaths in Taiwan [1]. OSCC development is a multistep process requiring the accumulation of multiple genetic alterations, influenced by a patient's genetic predisposition and by environmental factors. The environmental factors include alcohol and tobacco consumption, betel (areca) quid chewing, chronic inflammation, and viral infection [1]–[6]. Gene expression is affected by a single-nucleotide polymorphism (SNP) located within the promoter or other regulatory regions of the gene and are thought to be associated with the development of certain diseases [7]. To elucidate the complex process of carcinogenesis and improve the scientific basis for preventive interventions, the identification of major genes influencing a patient's susceptibility to OSCC should be prioritized.

Activation of nuclear factor-kappa B (NF-κB), a hallmark of the inflammatory response, is detected frequently in tumors and may play an important role in linking inflammation to tumor development and progression [8], [9]. NF-κB was originally identified as a nuclear factor specific to B cells bound to the B site of the κ-light chain gene enhancer. NF-κB is a heterodimer in the Rel family, which contains 5 members: RelA, RelB, c-Rel, p50/105 (NF-κB1), and p52/p100; the dimeric form of NF-κB p50/RelA is the most common form. NF-κB plays a central role in coordinating the expression of a wide variety of genes that control innate and adaptive immune responses, and also plays a critical role in cancer development and progression [8]. In normal cells, NF-κB is inactivated in the cytoplasm by binding to its inhibitor IκB. When IκB proteins are phosphorylated and degraded, NF-κB is released and further translocated to the nucleus, where gene transcription is initiated [10].

The IκB family includes IkappaB alpha (IκBα), IκBβ, IκBγ, IκBδ, IκBε, IκB-ζ, IκB-R, Bcl-3, p100, and p105, which are all constitutively expressed except for IκBα, which is inducibly expressed. IκBα is a classic form of the IκB family that can be found in the cytoplasm and nuclei [11]. Previous studies demonstrated that NF-κB suppression in cancer inhibits cell proliferation, causes cell-cycle arrest, and results in apoptosis, suggesting that NF-κB plays an important role in cell proliferation and survival [12]. Furthermore, NF-κB is known to prevent apoptosis by inducing antiapoptotic proteins, and suppresses the apoptotic potential of chemotherapeutic agents, leading to chemoresistance [13]. Aberrant expression of NF-κB proteins has been well documented in several types of cancer, including OSCC [14], with the level increasing gradually from premalignant lesions to invasive cancer [15]. Moreover, NF-κB1, which comprises p50 homodimers, transcriptionally regulates the antiapoptotic protein Bcl-2, which is overexpressed in a high proportion of oral cancer cases [16]. These various findings suggest that NF-κB signaling plays an important role in oral carcinogenesis.

Prior research has reported that polymorphic variations in promoter regions of the NF-κB1 gene *NFKB1* and the IκBα gene *NFKBIA* and in the 3′-untranslated region (3′-UTR) of *NFKBIA* were associated with a risk for Hodgkin's lymphoma, multiple myeloma, breast cancer, prostate cancer, gastric cancer, colorectal cancer, and melanoma [17]–[23]. In Taiwan Lin et al. investigated the −94 ins/del ATTG polymorphism in the *NFKB1* promoter among 3 population groups, namely, OSCC patients, male areca chewers with oral submucous fibrosis, and controls (non-areca chewers). They found no significant difference in *NFKB1* genotypes among the 3 groups [24]. However, after stratification by age, OSCC was shown to have a higher frequency of the insertion allelotype in patients older than 50 years. No significant difference was found for the *NFKB1* allelotype or genotype in patients with OSCC who exhibited different statuses of lymph node metastasis or clinical stage. Apart from the Lin et al. study, no reports have focused on the association between *NFKB1* and *NFKBIA* polymorphisms and OSCC development.

The current study investigated relationships between SNPs in the promoter regions of the *NFKB1* and *NFKBIA* genes and the risk of oral cancer. In addition, we evaluated the influences of these SNPs combined with betel nut and tobacco consumption, which lead to the susceptibility of oral cancer. We also investigated the relationship among genetic influences, environmental exposure, and the clinicopathological characteristics of oral cancer. To our knowledge, this is the first study to demonstrate a significant association between *NFKB1* and *NFKBIA* polymorphisms and oral carcinogenesis.

## Materials and Methods

### Subjects and specimen collection

We recruited 462 patients (444 men and 18 women, with a mean age of 54.4±11.4 years) at Chung Shan Medical University Hospital in Taichung and Changhua Christian Hospital and Show Chwan Memorial Hospital in Changhua, Taiwan. Patients were enrolled as a case group between 2007 and 2011. For the control group, we randomly chose 520 non-cancer people (426 men and 94 women, with a mean age of 52.4±14.7 years) who visited the same hospitals and resided in the same geographic area. Before commencement of the study, approval was obtained from the Institutional Review Board of Show Chwan Memorial Hospital, and each person provided written informed consent to participate in the study.

For both cases and controls, we used a questionnaire to obtain information on patient exposure to betel quid chewing, tobacco use, and alcohol consumption. Medical information for the cases was obtained from their medical records, and included TMN clinical staging, primary tumor size, lymph node involvement, and histologic grade. Oral cancer patients were clinically staged at the time of diagnosis according to the TNM staging system of the American Joint Committee on Cancer (AJCC) Staging Manual (7^th^ ed.) [25]. Tumor differentiation was examined by a pathologist according to the AJCC classification. Whole blood specimens collected from controls and OSCC patients were placed in tubes containing ethylenediaminetetraacetic acid (EDTA), and were immediately centrifuged and stored at −80°C.

### Genomic DNA Extraction

Genomic DNA was extracted using QIAamp DNA blood mini kits (Qiagen, Valencia, CA, USA) following the manufacturer's instructions. We dissolved DNA in a TE buffer (10 mM Tris, 1 mM EDTA; pH 7.8), which was subsequently quantified by measuring the OD260. The final preparation was stored at −20°C and used to create templates for the polymerase chain reaction (PCR).

### Real-time PCR

The allelic discrimination of the *NFKB1* −94 ATTG, *NFKBIA* −519, *NFKBIA* −826, and *NFKBIA* −881 gene polymorphisms were assessed with the ABI StepOne™ Real-Time PCR System (Applied Biosystems) and analyzed with SDS v3.0 software (Applied Biosystems) using the TaqMan assay. The final volume for each reaction was 5 µL, containing 2.5 µL TaqMan Genotyping Master Mix, 0.125 µL TaqMan probes mix, and 10 ng genomic DNA. The real-time PCR included an initial denaturation step at 95°C for 10 min, followed by 40 cycles at of 95°C for 15 s and then at 60°C for 1 min.

### Statistical analysis

Differences between the 2 groups were considered significant if *p*-values were less than .05. Hardy-Weinberg equilibrium (HWE) was assessed using a goodness-of-fit *X^2^*-test for biallelic markers. The Mann-Whitney *U*-test and Fisher's exact test were used to compare differences in the distributions of patient demographic characteristics between the non-cancer (control) and oral cancer groups. The adjusted odds ratios (ORs) and 95% confidence intervals (CIs) of the association between genotype frequencies and risk in addition to clinicopathological characteristics were estimated using multiple logistic regression models, after controlling for other covariates. We analyzed all data using the Statistical Analytic System (SAS Institute, Cary, NC, USA) software (v. 9.1, 2005) for Windows.

## Results

The statistical analysis of demographic characteristics is shown in Table 1. We found significantly different distributions of age (*p* = 0.023), sex (*p*<0.001), betel quid chewing (*p*<0.001), alcohol consumption (*p*<0.001), and tobacco use (*p*<0.001) between control participants and OSCC patients. To reduce the possible interference of environmental factors, the adjusted ORs (AORs) with 95% CIs were estimated by multiple logistic regression models after controlling for other covariates in each comparison.

**Table 1:** Distributions of demographic characteristics in 520 controls and 462 patients with oral cancer.

Variable	Controls (N = 520)	Patients (N = 462)	*P*-value
**Age (y)**	**Mean ± S.D.**	**Mean ± S.D.**	
	52.4±14.7	54.4±11.4	*p* = .023*
**Sex**	**n (%)**	**n (%)**	
Male	426 (81.9%)	444 (96.1%)	
Female	94 (18.1%)	18 (3.9%)	*p*<.0001*
**Betel nut chewing**			
No	429 (82.5%)	109 (23.6%)	
Yes	91 (17.5%)	353 (76.4%)	*p*<.0001*
**Alcohol consumption**			
No	309 (59.4%)	177 (38.3%)	
Yes	211 (40.3%)	285 (61.7%)	*p*<.0001*
**Tobacco consumption**			
No	310 (59.6%)	74 (16.0%)	
Yes	210 (40.4%)	388 (84.0%)	*p*<.0001*

Mann-Whitney U test or Fisher's exact test were used to analyze differences between controls and patients with oral cancer.

* indicates statistical significance at *p*<0.05.

For the control group, all analyzed gene markers were in HWE (*p*>0.05). The data in Table 2 show that, for both OSCC patients and the controls, alleles with the highest distribution frequency were as follows: heterozygous ATGG1/ATGG2 for the −94 locus of the *NFKB1* gene; and homozygous C/C, C/C, and AA, respectively, for −519, −826, and −881 loci of the *NFKBIA* gene. According to the AORs (95% CI), significantly (*p*<0.05) higher risks for OSCC were noted for *NFKB1* gene polymorphisms −94 ATGG1/ATGG2,−94 ATGG2/ATGG2, and the combination of −94 ATGG1/ATGG2 and ATGG2/ATGG2 genotypes. Compared with the corresponding wild-type (WT) homozygotes of the control group, the risk for OSCC was 1.8-fold (95% CI = 1.2∼2.8) for −94 ATGG1/ATGG2; 2.2-fold (95% CI = 1.2–4.2) for −94 ATGG2/ATGG2l; and 1.8-fold (95% CI = 1.2–2.8) for the combination of −94 ATGG1/ATGG2 and ATGG2/ATGG2. These results were obtained after adjusting for age, sex, betel quid chewing, alcohol consumption, and tobacco use.

**Table 2:** Distribution frequencies for *NFKB* and *NFKBIA* genotypes in 520 controls and 462 oral cancer patients.

Variable	Controls (N = 520) n (%)	Patients (N = 462) n (%)	OR (95% CI)	AOR (95% CI)
**NFkB1**				
Del/Del	168 (32.3%)	100 (21.6%)	1.0	1.0
Del/Ins	271 (52.1%)	246 (53.3%)	**1.5 (1.1–2.1)**	**1.8 (1.1–2.7)**
Ins/Ins	81 (15.6%)	116 (25.1%)	**1.5 (1.1–2.1)**	**2.2 (1.2–4.2)**
Del/Ins+Ins/Ins	352 (67.7%)	362 (78.4%)	**1.7 (1.3–2.3)**	**1.8 (1.2–2.8)**
**NFKBIA −519**				
CC	432 (83.1%)	381 (82.5%)	1.0	1.0
CT	86 (16.5%)	78 (16.9%)	1.0 (0.7–1.4)	1.3 (0.9–2.2)
TT	2 (0.4%)	3 (0.6%)	1.7 (0.3–10.2)	5.4 (0.4–74.1)
CT+TT	88 (16.9%)	81 (17.5%)	1.0 (0.7–1.5)	1.4 (0.9–2.2)
**NFKBIA −826**				
CC	438 (84.2%)	351 (76.0%)	1.0	1.0
CT	78 (15.0%)	101 (21.8%)	**1.6 (1.2–2.2)**	**1.6 (1.0–2.6)**
TT	4 (0.8%)	10 (2.2%)	3.1 (1.0–10.0)	3.4 (0.6–18.3)
CT+TT	82 (15.8%)	111 (24.0%)	**1.7 (1.2–2.3)**	**1.7 (1.2–2.7)**
**NFKBIA −881**				
AA	438 (84.2%)	351 (76.0%)	1.0	1.0
AG	78 (15.0%)	101 (21.8%)	**1.6 (1.2–2.2)**	**1.6 (1.0–2.6)**
GG	4 (0.8%)	10 (2.2%)	3.1 (1.0–10.0)	3.4 (0.6–18.3)
AG+GG	82 (15.8%)	111 (24.0%)	**1.7 (1.2–2.3)**	**1.7 (1.2–2.7)**

Odds ratios (ORs) and their 95% confidence intervals (CIs) were estimated by logistic regression models. The adjusted odds ratios (AORs) with 95% CIs were estimated by multiple logistic regression models after controlling for age, sex, betel quid chewing, alcohol consumption, and tobacco use.

We also compared the results from participants with the polymorphic gene to those from participants showing the WT gene. We found significantly higher risks for OSCC in participants with the *NFKBIA* −826 C/T, −826 C/T+T/T, −881 A/G, and −881 A/G+GG polymorphic genotypes. These risks were, respectively, 1.6-fold (95% CI = 1.0–2.6), 1.7-fold (95% CI = 1.1–2.7), 1.6-fold (95% CI = 1.0–2.6), and 1.7-fold (95% CI = 1.2–2.7), compared with the WT. However, people with the *NFKBIA* −519 polymorphic gene were not at a significantly higher risk than those with the WT gene.

Interactive effects between environmental risk factors and genetic polymorphisms of *NFKB1* and *NFKBIA* are shown in Tables 3 and 4. Among 598 smokers, participants with at least one ATGG2 allele of *NFKB1 −*94, the T allele of either *NFKBIA −*519 or *−*826, or the G allele of *NFKBIA −*881 were at a higher risk for OSCC if they also chewed betel nut. These increased risks were 67.3-fold (95% CI = 18.9–239.4), 29.5-fold (95% CI = 9.6–91.1), 45.0-fold (95% CI = 14.6–138.5), and 45.0-fold (95% CI = 14.6–138.5), respectively. Similarly, compared with people who had WT homozygotes but did not chew betel nut, people who chewed betel nut and had any one of these polymorphisms were at higher risk for OSCC development. These increased risks were as follows: 6.9-fold (95% CI = 2.5–18.9) for the ATGG2 allele of *NFKB1 −*94; 14.2-fold (95% CI = 8.3–24.5) for the T allele of *NFKBIA* −519; 12.4-fold (95% CI = 7.3–21.2) for the T allele of *NFKBIA −*826; and 12.4-fold (95% CI = 7.3–21.2) for the G allele of *NFKBIA −*881 (Table 3).

**Table 3:** Adjusted odds ratio (AOR) and 95% CI for oral cancer associated with *NFKB* and *NFKBIA* genotypic frequencies and betel nut chewing among 598 smokers.

Variable	Controls (n = 210) (%)	Patients (n = 388) (%)	OR (95% CI)	AOR (95% CI)
**NFKB1**				
aDel/Del genotype & non-betel nut chewing	39 (18.6%)	7 (1.8%)	1.0	1.0
bDel/Ins or Ins/Ins genotype or betel nut chewing	118 (56.2%)	128 (33.0%)	**6.0 (2.6–14.0)**	**6.9 (2.5–18.9)**
cDel/Ins or Ins/Ins genotype with betel nut chewing	53 (25.2%)	253 (65.2%)	**26.6 (11.3–62.7)**	**67.4 (18.9–239.4)**
**NFKBIA −519**				
aCC genotype & non-betel nut chewing	119 (56.7%)	47 (12.1%)	1.0	1.0
bCT or TT genotype or betel nut chewing	81 (38.6%)	290 (74.8%)	**9.1 (6.0–13.8)**	**14.2 (8.3–24.5)**
cCT or TT genotype with betel nut chewing	10 (4.7%)	51 (13.1%)	**12.9 (6.1–27.5)**	**29.5 (9.6–91.1)**
**NFKBIA −826**				
aCC genotype & non-betel nut chewing	123 (58.6%)	20 (13.1%)	1.0	1.0
bCT or TT genotype or betel nut chewing	76 (36.2%)	134 (65.5%)	**8.1 (5.3–12.2)**	**12.4 (7.3–21.2)**
cCT or TT genotype with betel nut chewing	11 (5.2%)	138 (21.4%)	**18.2 (9.0–37.0)**	**45.0 (14.6–138.5)**
**NFKBIA −881**				
aAA genotype & non-betel nut chewing	123 (58.6%)	20 (13.1%)	1.0	1.0
bAG or GG genotype or betel nut chewing	76 (36.2%)	134 (65.5%)	**8.1 (5.3–12.2)**	**12.4 (7.3–21.2)**
cAG or GG genotype with betel nut chewing	11 (5.2%)	138 (21.4%)	**18.2 (9.0–37.0)**	**45.0 (14.6–138.5)**

ORs with 95% CIs were estimated by logistic regression models. AORs with 95% CIs were estimated by multiple logistic regression models after controlling for age and sex.

a People with wild genotype but not chewers of betel nut.

b People with either one or more mutated genotypes, or chewers of betel nut (but not both).

c People with at least one mutated genotype and also chewers of betel nut.

**Table 4:** Adjusted odds ratio (AOR) and 95% CI for oral cancer associated with *NFKB* and *NFKBIA* genotypic frequencies and cigarette smoking among 444 betel nut consumers.

Variable	Controls (n = 91) (%)	Patients (n = 353) (%)	OR (95% CI)	AOR (95% CI)
**NFKB1**				
aDel/Del genotype & non-smoker	8 (8.8%)	7 (2.0%)	1.0	1.0
bDel/Ins or Ins/Ins genotype or smoker	30 (33.0%)	93 (26.3%)	3.5 (1.2–10.6)	3.0 (0.7–13.8)
cDel/Ins or Ins/Ins genotype with smoking	53 (58.2%)	253 (71.7%)	**5.5 (1.9–15.7)**	**6.9 (1.7–28.6)**
**NFKBIA −519**				
aCC genotype & non-smoker	16 (17.6%)	18 (5.1%)	1.0	1.0
bCT or TT genotype or smoker	65 (71.4%)	284 (80.5%)	**3.9 (1.9–8.0)**	**5.9 (2.3–15.0)**
cCT or TT genotype with smoking	10 (11.0%)	51 (14.4%)	**4.5 (1.7–11.8)**	**14.5 (2.0–106.0)**
**NFKBIA −826**				
aCC genotype & non-smoker	15 (16.5%)	19 (5.4%)	1.0	1.0
bCT or TT genotype or smoker	65 (71.4%)	251 (71.1%)	**3.0 (1.5–6.3)**	**4.7 (1.9–11.6)**
cCT or TT genotype with smoking	11 (12.1%)	83 (23.5%)	**6.0 (2.4–15.0)**	**13.9 (3.2–59.8)**
**NFKBIA −881**				
aAA genotype & non-smoker	15 (16.5%)	19 (5.4%)	1.0	1.0
bAG or GG genotype or smoker	65 (71.4%)	251 (71.1%)	**3.0 (1.5–6.3)**	**4.7 (1.9–11.6)**
cAG or GG genotype with smoking	11 (12.1%)	83 (23.5%)	**6.0 (2.4–15.0)**	**13.9 (3.2–59.8)**

Odds ratios (ORs) with their 95% confidence intervals were estimated by logistic regression models.

Adjusted odds ratios (AORs) with their 95% confidence intervals were estimated by multiple logistic regression models after controlling for age and sex.

a People with wild genotype but not cigarette smokers.

b People with either one or more mutated genotypes, or smokers (but not both).

c People with at least one mutated genotype and also cigarette smokers.

Among betel nut consumers in the cohort, tobacco smoking elevated oral cancer risk significantly in participants polymorphic for *NFKB1* in the *−*94 locus or *NFKBIA* in three loci (*−*519, *−*826, and *−*881), compared with people with the WT gene who did not smoke tobacco (Table 4). Moreover, people who were either polymorphic for *NFKBIA* in 3 loci (*−*519, *−*826, and *−*881) or who smoked were at 4.67- to 5.91-fold risk (*p*<0.05) of developing oral cancer, compared with people with the WT gene who did not smoke (Table 4). These results suggest that *NFKB1* and *NFKBIA* gene polymorphisms exert a strong influence on oral cancer susceptibility in men who chew betel nut and/or smoke tobacco.

To explore the effects of polymorphic genotypes of *NFKB1* and *NFKBIA* on the clinical status of OSCC, we classified OSCC patients into 2 subgroups. In the first subgroup, patients had homozygous WT alleles, and in the second subgroup, they had at least one polymorphic allele. For the genotypic frequencies of the SNPs, only *NFKBIA* −519 showed a significant association with clinical pathological variables in OSCC patients. Compared with the WT genotype (C/C), patients with at least one polymorphic T allele of *NFKBIA* −519 showed a higher risk (4.88-fold; 95% CI = 1.2–20.4) for distant metastasis (Table 5).

**Table 5:** Distribution frequencies for clinical status and *NFKBIA*−519 genotype in 462 patients with OSCC.

Variable	genotypic frequencies			
	CC (N = 381) n (%)	CT+TT (N = 81) n (%)	OR (95% CI)	AOR (95% CI)
Clinical Stage				
Stage I/II	174 (45.7%)	39 (48.1%)	1.0	1.0
Stage III/IV	207 (54.3%)	42 (51.9%)	0.9 (0.6–1.5)	0.7 (0.4–1.3)
Tumor size				
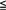 T2	241 (63.3%)	52 (64.2%)	1.0	1.0
>T2	140 (36.7%)	29 (35.8%)	1.0 (0.6–1.6)	0.7 (0.4–1.3)
Lymph node metastasis				
No	246 (64.6%)	52 (64.2%)	1.0	1.0
Yes	135 (35.4%)	29 (35.8%)	1.0 (0.6–1.7)	1.1 (0.6–1.9)
Distant metastasis				
No	377 (99.0%)	77 (95.1%)	1.0	1.0
Yes	4 (1.0%)	4 (4.9%)	**4.9 (1.2–20.0)** *	**4.9 (1.2–20.4)** *
Cell differentiation				
Well differentiated	56 (14.7%)	12 (18.8%)	1.0	1.0
Moderately or poorly differentiated	325 (85.3%)	69 (85.2%)	1.0 (0.5–1.9)	1.1 (0.5–2.4)

ORs with 95% CIs were estimated by logistic regression models.

AORs with 95% CIs were estimated by multiple logistic regression models after controlling for sex, betel quid chewing, alcohol consumption, and tobacco consumption.

>T2: tumor size >2 cm in the greatest dimension.

* indicates statistical significance at *p*<0.05.

## Discussion

The NF-κB pathway plays an important role in tumor development and aggressiveness by enhancing tumor angiogenesis, antiapoptosis, and proliferation, and by repressing the immune response [12], [26]. NF-κB is rarely found to be constitutively active in normal cells, but it is constitutively active in most tumor cell lines [27]–[29]. Prior research has demonstrated that NF-κB is constitutively activated in OSCC [15]. Furthermore, the −94 insertion/deletion ATTG polymorphism was shown to have a regulatory influence on *NFKB1* gene expression; the promoter sequence containing the ATTG2 allele displayed a 2× higher activity than comparable sequences containing the ATTG1 allele [30]. The allele plays an important role in susceptibility to prostate cancer, cervical squamous cell carcinoma, gastric cancer, and hepatocellular carcinoma [22], [23], [31], [32]. Thus, increased risk for oral cancer associated with the *NFKB1* ATGG1/ATGG2 variant might have resulted from its positive regulation of NF-κB expression (Table 2).

Alcohol consumption, tobacco smoking, and betel quid chewing are the main known etiologic factors of oral cancer. In this study, we observed that higher ratios of people in the OSCC group had consumed alcohol and tobacco and chewed betel quid (61.7%, 84.0%, and 76.4%, respectively), compared with the controls (40.3%, 40.4%, and 17.5%, respectively) (Table 1). This finding indicated that alcohol and tobacco consumption and betel quid chewing are highly associated with increased risks for oral cancer. Long-term tobacco smoking and betel nut chewing have been shown to contribute to carcinogenesis [3]–[5], [33]. Betel nut constituents can increase protein levels of c-fos and c-jun proto-oncogenes, and tobacco consumption can significantly increase nuclear hypoxia-inducible factor (HIF)-1α expression in oral cancer [34], [35].

People carrying the *murine double minute 2 (MDM2)* SNP 309 GG genotype have oral mucosa that is more susceptible to environmental carcinogens, including tobacco, alcohol, and betel nuts [36]. Therefore, exposure to these factors can result in earlier onset of tumor formation [36]. Accordingly, we evaluated the combined effect of environmental carcinogens and *NFKB1* gene polymorphisms on the risk of oral cancer (Tables 2, 3). We found that the *NFKB1* −94 insertion/deletion ATTG polymorphism combined with betel quid chewing and smoking further increased risk of oral carcinogenesis. This phenomenon might be caused by alterations in the binding affinities between betel nut and tobacco constituents and the promoter of the polymorphic *NFKB1* gene. Consequently, expression or activity of NF-κB was further changed. Several earlier reports have indicated that tobacco and betel nut constituents can induce NF-κB activation in oral keratinocytes [37], [38]. Moreover, evidence has shown that alkaline saliva generated by chewing betel quid may play a role in cigarette-related nicotine-induced DNA damage, and reactive oxygen species may be involved in generating this DNA damage [39]. These findings provide a possible molecular explanation for the synergistic effect of betel quid chewing and smoking in oral cancer development. However, details underlying the mechanism must be verified by other well-designed experiments.

An imbalance between NF-κB and IκB is a critical step in tumor development and response to treatment [40]. Polymorphisms of the 3 loci (*−*519, *−*826, and *−*881) of the *NFKBIA* promoter were investigated in this study. The −881 position was selected based on potential functional effects because of its location within the transcription factor, retinoic acid-related orphan receptor α (RORα), and binding sites [41]. *NFKBIA* −881G might alter the binding ability of RORα, which plays a potential role in cancer development [41]. Moreover, *−*881 and −826 in the *NFKBIA* promoter showed strong local linkage disequilibrium (LD) in 2 Asian populations (Chinese and Japanese). The current study findings showed that Taiwanese men with at least one polymorphic G allele of *NFKBIA* −881 or the T allele of *NFKBIA* −826 are at a high risk for oral carcinogenesis (Table 2). The synergistic effect of environmental factors (betel quid and smoking) and *NFKBIA* −881 and −826 polymorphisms on the risk of oral cancer was also demonstrated adequately (Tables 3 and 4).

Although to date no direct evidence has been presented proving that −881G or −826T at the promoter of *NFKBIA* decreases promoter activity, indirect evidence indicates this possibility. For instance, NF-κB expression was twice as high in patients with sarcoidosis, compared with that of control participants. In contrast, the −881G and −826T allelic carriages were more prevalent in sarcoidosis patients than in controls [42], indicating that the −881G and −826T allelic carriages might be associated with increased activity of NF-κB. Because IκBα is an absolute requirement for normal termination of the NF-κB response [11], *NFKBIA* −881G and −826T allelic carriages were thought to retard efficient IκBα expression.

In conclusion, our results suggest that *NFKB1* gene polymorphisms might be correlated with oral cancer susceptibility, and the combined effect of *NFKB1* or *NFKBIA* gene polymorphisms with environmental carcinogens increases risk of oral cancer development significantly. Patients with oral cancer carrying at least one T allele of *NFKBIA* −519 are at higher risk of developing distal metastasis, compared with patients carrying C/C homozygotes.
